# Isolation and Molecular Identification of Pathogenic Free-Living Amoebae from Environmental Samples in Tenerife, Canary Islands, Spain

**DOI:** 10.1021/acsestwater.4c00573

**Published:** 2025-05-02

**Authors:** Patricia Pérez-Pérez, María Reyes-Batlle, Rodrigo Morchón, José E. Piñero, Jacob Lorenzo-Morales

**Affiliations:** † Instituto Universitario de Enfermedades Tropicales y Salud Pública de Canarias (IUETSPC), 16749 Universidad de La Laguna (ULL), Avenida Astrofísico Francisco Sánchez S/N, San Cristóbal de La Laguna, 38206 Tenerife, Spain; ‡ Departamento de Obstetricia y Ginecología, Pediatría, Medicina Preventiva y Salud Pública, Toxicología, Medicina Legal y Forense y Parasitología, Facultad de Farmacia, Universidad de La Laguna, San Cristóbal de La Laguna 38200 Tenerife, Spain; § Consorcio Centro de Investigación Biomédica en Red de Enfermedades Infecciosas (CIBERINFEC), Instituto de Salud Carlos III, Madrid 28029, Spain; ∥ Zoonotic Infections and One Health GIR, Laboratory of Parasitology, Faculty of Pharmacy, University of Salamanca, Salamanca 37008, Spain

**Keywords:** Tenerife, soil, water, *Acanthamoeba* spp., *Vermamoeba vermiformis*, environmental monitoring

## Abstract

Free-living amoebae (FLA) are protozoa that have been reported worldwide from various environmental sources. However, only some groups (Acanthamoeba spp., Naegleria fowleri, Balamuthia mandrillaris, Sappinia pedata, Vahlkampfia spp., and Vermamoeba vermiformis) are considered to be pathogenic for humans and other animals. FLA has been previously reported in the Canary Islands archipelago since 2005. However, climate change and other environmental factors that could affect the communities of FLA in the islands lead to the development of this extra study. This study was carried out in Tenerife, the largest and most populated island, between June 2021 and July 2022. A total of 28 soil samples and 23 water samples were seeded in 2% non-nutrient agar (NNA) plates, incubated at 26 °C, and monitored daily to evaluate the presence of FLA. DNA was extracted from those plates on which there was suspected FLA growth, and polymerase chain reaction amplification of the 18S rRNA gene (DF3• region on the case of Acanthamoeba) was carried out. The obtained results showed that the genus Acanthamoeba (25/51:48.07%), with the genotype T4 being the most common (21/25; 84%), and the species V. vermiformis (7/51; 13.73%) were the most abundant FLA in the surveyed samples collected from Tenerife.

## Graphical abstract

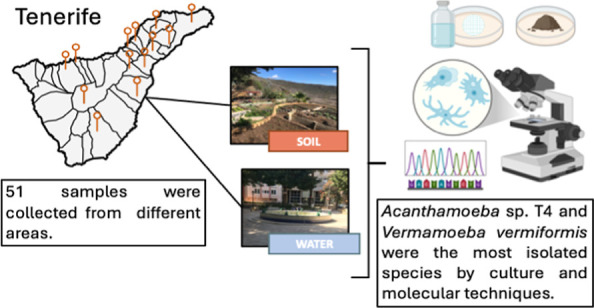

## Introduction

The Canary Islands constitute an archipelago of eight inhabited major islands and six islets, located in the Atlantic Ocean, between latitudes 27°38′ and 29°25′ North (which is more than 200 km from North to South) and longitudes 13°30′ and 18°19′ West (500 km from East to West, approximately). This adds up to 7499 km^2^ in total, with volcanic origin between 21 and 0.8 million years old (Myr).^1,2^ Due to the cool current of the subtropical North Atlantic and the influence of the northeast trade winds, the climate in the Canary Islands is mild. Occasionally, as the cool trade wind wanes, easterly Saharan air with high temperatures and suspended desert dust arrives to the Canaries, causing the relative humidity drop.^3^

Free-living amoeba (FLA) are aerobic and mitochondriate eukaryotic protozoa, widely distributed in nature and human-related environments. They have been found in water, soil, and air, but also in sewage, swimming pools, flowerpots, water tubs, humidifiers, aquaria, eye wash solutions, and hospital environments, e.g., dialysis and dental treatment units.^4^ Despite having the ability to live endozoically, these species are known as amphizoid amoebae since they can also live without a host.^5^

From many genera of FLA that exist in nature, Acanthamoeba spp., Naegleria fowleri, Sappinia pedata, Balamuthia mandrillaris, Vermamoeba vermiformis, and Vahlkampfia spp. are involved in human and other animal infections. Regarding brain infections, N. fowleri is the causative agent of primary amoebic meningoencephalitis, whereas Acanthamoeba spp., B. mandrillaris and S. pedata cause granulomatous amoebic encephalitis (GAE).^5–7^ Acanthamoeba is not the only cause of amoebic keratitis, because this condition may also be caused by other FLA, such as V. vermiformis and vahlkampfiid amoebae.^8^ Thus, V. vermiformis has been identified as both an etiological agent and a pathogen reservoir and, according to a recent study, it is the cause of a painful ulcer next to the eye.^9^ Besides, B. mandrillaris and Acanthamoeba spp. cause dermatitis in immunosuppressed individuals.^5^

The presence of FLA has been reported in 5 of the 8 major Canary Islands. Among the Eastern islands, Fuerteventura was the one where a wider variety of FLA types have been found in soil and water, such as the Acanthamoeba and Naegleria genera and the V. vermiformis species.^10,11^ On the other hand, in Lanzarote, only the presence of the thermotolerant amoeba V. vermiformis was detected in water samples.^12^ However, in Gran Canaria, the presence of Acanthamoeba species was reported in the soils of 8 from the 21 municipalities.^13^ Among the Western islands, the presence of the two potentially pathogenic FLA V.vermiformis^14^ and Acanthamoeba spp.^15^ was seen in the islands of El Hierro. However, the island of Tenerife has been the most sampled over time, isolating V.vermiformis from El Teide Mount snow,^16^ Acanthamoeba spp. from several water and soil sources,^17,18^ and Naegleria spp. from a recreational fountain in the capital city.^19^

Amoebic diseases are difficult to diagnose, which leads to delayed treatment and underreporting of amoebic infection cases globally, resulting in a high mortality rate.^20^ Therefore, the aim of this research is to carry out a control and follow-up study of FLA in environmental samples in the most populated island of the Canary Islands, Tenerife.

## Materials and Methods

### Location and Sampling

The island of Tenerife, whose province Santa Cruz de Tenerife is one of the capitals of the Canary Islands archipelago, has an area of 2034.38 km^2^, which makes it the largest island. With 927,993 inhabitants, it is the most populated among the eight Canary Islands^21^ (Figure 1).

**Figure 1:**
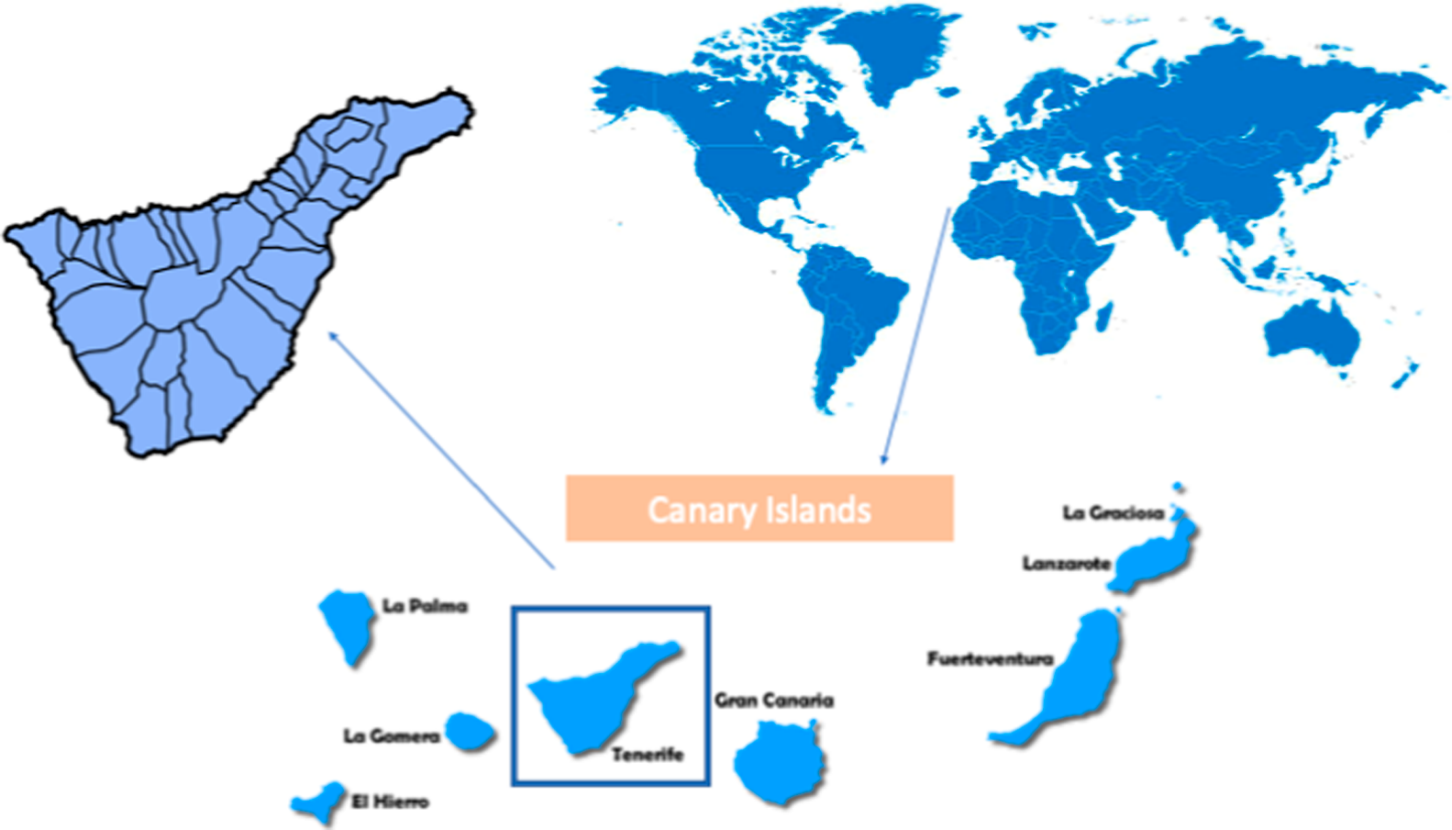
Geographical localization of Tenerife Island.

A total of 51 samples were collected from several locations in Tenerife, Canary Islands (28°16′07″N 16°36′20″O) during the years 2021 and 2022 in order to detect the presence of potentially pathogenic FLA in water and soil samples.

#### Water Samples

A total of 23 water samples were collected in sterile bottles (1 L), which correspond to a recreational fountain (9/23), tap from a private home (11/23), ravine of a small stream that runs through it (1/23), fish tank of freshwater from a decorative tank in a home (1/23), and irrigation water (1/23).

#### Soil Samples

28 soil samples were taken in a 15 mL sterile tube from school gardens (15/28) and vegetable patches from private gardens (13/28). They are not irrigated with potable water but with irrigation water that comes from reservoirs or artificial canals and is channeled for use in agricultural activities, such as orchard irrigation. Samples were stored at room temperature until they underwent additional laboratory processing (Figure 2).

**Figure 2:**
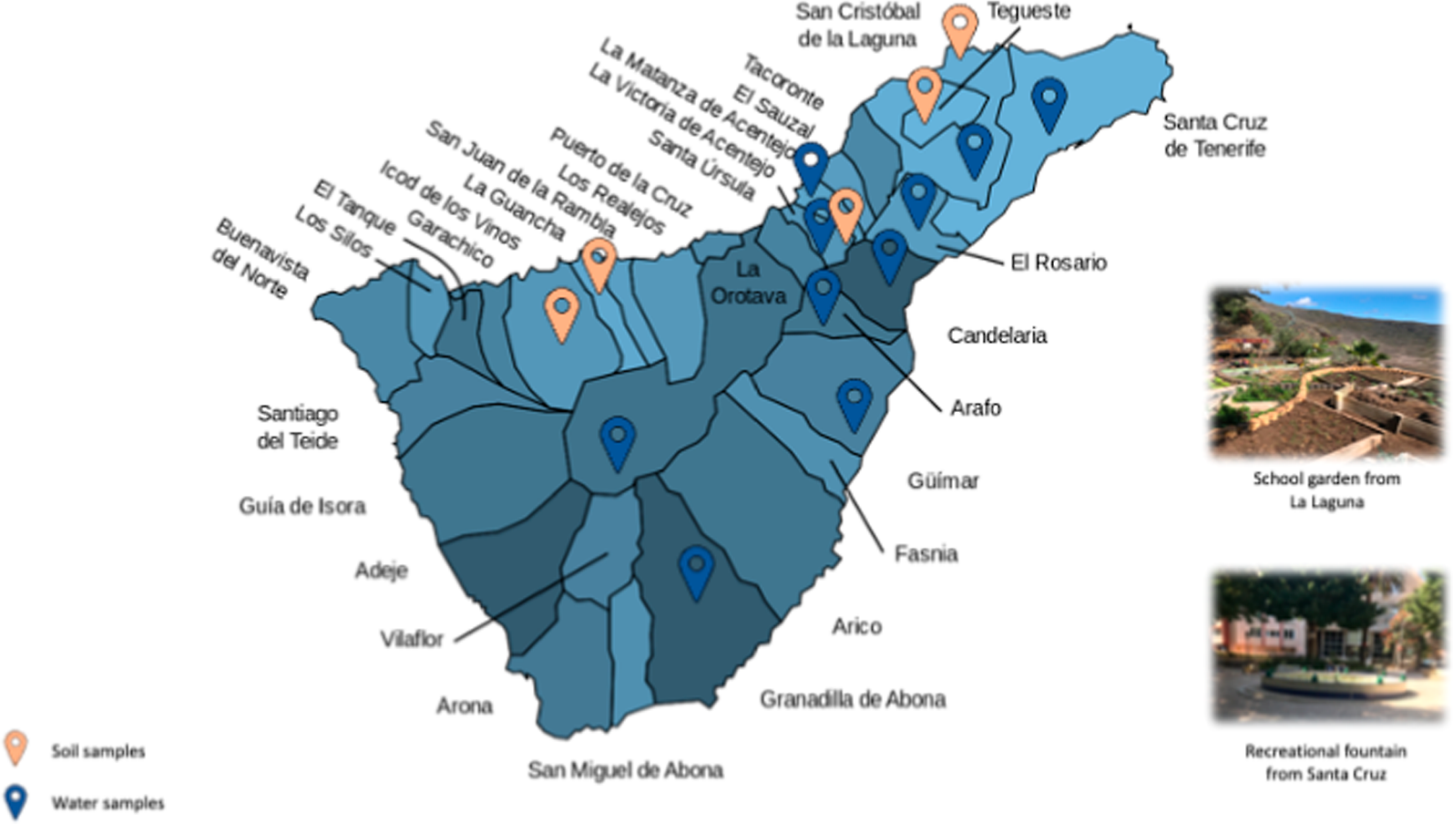
Sampling sites of soil and water in Tenerife.

### Free-Living Amoeba Isolation

A total of 1 L of each water sample was filtered by a vacuum filtration system using a 0.45 μm pore size filter (Pall, Madrid, Spain). The membrane filters were then inverted onto non-nutrient agar (NNA) plates with a layer of heat-killed Escherichia coli. Moreover, 0.5 g of each soil sample was seeded directly onto an NNA plate, performing a soil-striation along the Petri dish. The plates were incubated at room temperature and monitored every day for amoebic growth for up to 15 days with an inverted microscope. In those plates where FLA were present, they were cloned by dilution in a new NNA until a monoaxenic culture was obtained when possible.^12^ This consists of marking an area with amoebae as far away from fungi or other protozoa as possible and cutting out the marked piece of agar from the original plate and transferring it, face down, to a new plate with NNA so that the amoebae move to the surface of the new agar. This process was repeated as many times as necessary, until the amoeba was isolated. Prior to using molecular techniques, isolated FLA were classified at the genus level based on their morphology using Page’s criteria.^22^

### DNA Extraction

DNA from positive samples was extracted from 1 to 2 mL of amoebic culture suspension for molecular characterization. To obtain this amoeba suspension, 4 mL of Page’s Amoeba Solution was added to the plate with the monoxenic amoeba culture. The plate was scraped with a glass spoon to create the amoeba suspension. The suspension was centrifuged, and the concentrated amoeba culture was directly placed into the Maxwell 16 tissue DNA purification kit sample cartridge (Promega, Madrid, Spain) as it has been described before^23^ and following the manufacturer’s instructions. Extracted DNA yield and purity was quantified using a DS-11 spectrophotometer (DeNovix, USA).

### PCR and Molecular Characterization of Isolates

Extracted DNAs were stored at −20 °C until their use as a template for polymerase chain reaction (PCR) amplification, using different primer sets: universal FLA-f 5′- CGCGGTAATTCCAGCTCCAATAGC −3′/FLA-r5′-CAGGTTAAGGTCTCGTTCGTTAAC-3′^24^ and JDP-1f 5′-GGCCCAGATCGTTTACCGTGAA-3′ and JDP-2r5′-TCTCACAAGCTGCTAGGGAGTCA-3′ for cells that resemble Acanthamoeba spp.,^25^ and for the family Vahlkampfiidae, these primers were used: VAHL1 5′-GTCTTCGTAGGTGAACCTGC-3′ and VAHL2 5′-CCGCTTACTGATATGCTTAA-3′.^26^ For all performed PCRs, amplification reactions were carried out in a 50 μL mixture containing 80 ng DNA for FLA, 60 ng DNA for VAHL, and 40 ng DNA for Acanthamoeba spp., and the conditions for the PCRs shown in the table were established (Table 1). Considering the use of different amounts of DNA for PCR conditions with universal and specific primers to optimize sensitivity, specificity, and amplification efficiency, more DNA is needed for universal primers due to the need to detect multiple species in a potentially diverse environment, while less DNA is sufficient for specific primers as these are designed to amplify a single target with high specificity. This strategy is common in PCR studies to ensure accurate and reliable results. DNA was visualized with 2% agarose gel stained with Gel Red. Amplicons were sent to the commercial sequencing company Macrogen, Spain) for sequencing and identified with a GenBank ID from the National Library of Medicine (NCBI).

**Table 1:** PCR Conditions for the Different Primers

primer sets	PCR’s conditions				
	initiation	denaturation	annealing	primer extension	extension
FLA-f/r (24)	95 °C,2′	95 °C,30″	55 °C,30″	72 °C,30″	72 °C,7′
		40 cycles			
JDP-1*f*/2r(25)	95 °C,2′	95 °C,30″	50 °C,30″	72 °C,30″	72 °C,7′
		35 cycles			
VAHL-1/2(26)	95 °C,2′	95 °C,30″	50 °C,30″	72 °C,30″	72 °C,7′
		35 cycles			

### Phylogenetic Analysis

To establish the genetic correlation among the isolates, a sequence alignment was done using the MEGA11 tool.^27^ The neighbor-joining method was used to infer the evolutionary history.^28^ The maximum composite likelihood method was utilized to compute the evolutionary distances, which are expressed as base substitutions per site.

## Results

From the total of 51 samples, all soil samples (28/28; 100%) and 10 water samples were positive for the presence of FLA in NNA plates (10/23; 43.47%). After analysis of the 18S rRNA gene (the DF3 region in the case of Acanthamoeba), 27 soil samples (27/28; 96.43%) and 8 water samples (8/10; 80%) were positive for PCR. For PCR results, 8 amoebae were detected with the universal primers from the total number of samples analyzed, 25 amoebae using the specific JDP primers, and 2 with the Vahl primers. The amplicon length varies, at 800bp for FLA primers and 500bp for JDP and Vahl primers. Acanthamoeba spp. were the most abundantly isolated species in soils, with a total of 22 samples (22/28; 78.57%), with the T4 genotype being the most common (19/22; 86.36%) followed by T2 (2/22; 9.09%) and T3 genotype (1/22; 4.54%) (Table S2) (Figure 3).

**Figure 3:**
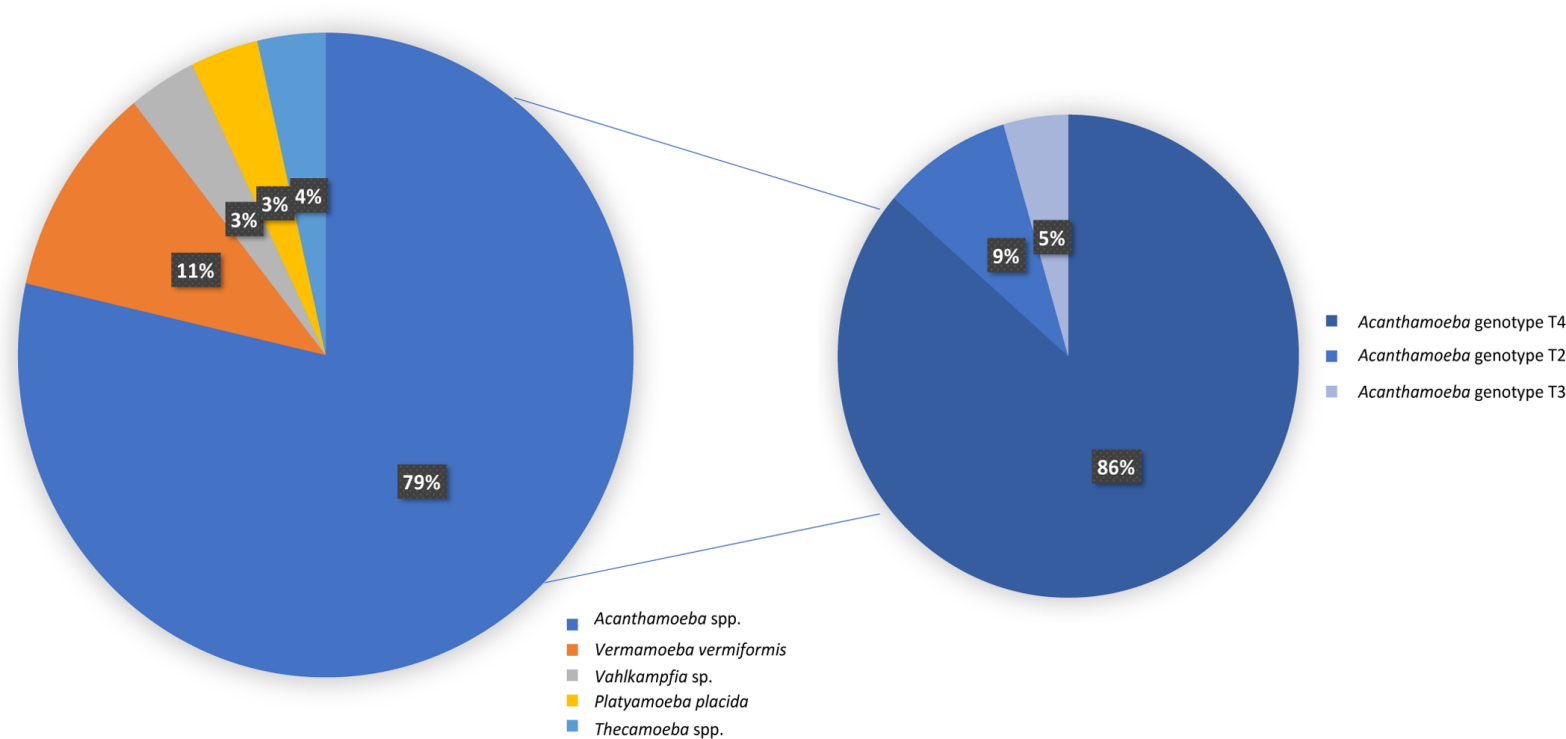
Distribution of FLA isolated in soil samples of Tenerife.

In contrast, the presence of V. vermiformis in the water samples was prominent (4/23; 17.39%). Acanthamoeba spp. was the second most frequently found (3/23; 13.04%), with the T4 genotype being the only one detected (3/3; 100%). Naegleria fultoni, Thecamoeba spp., and Cercozoa spp. were isolated in samples of TFEW1, TFEW2, and TFEW23, respectively, with a 4.35% prevalence for each of them in this study (1/23) (Table S3 and Figures 4,5, and[Fig fig6]).

**Figure 4:**
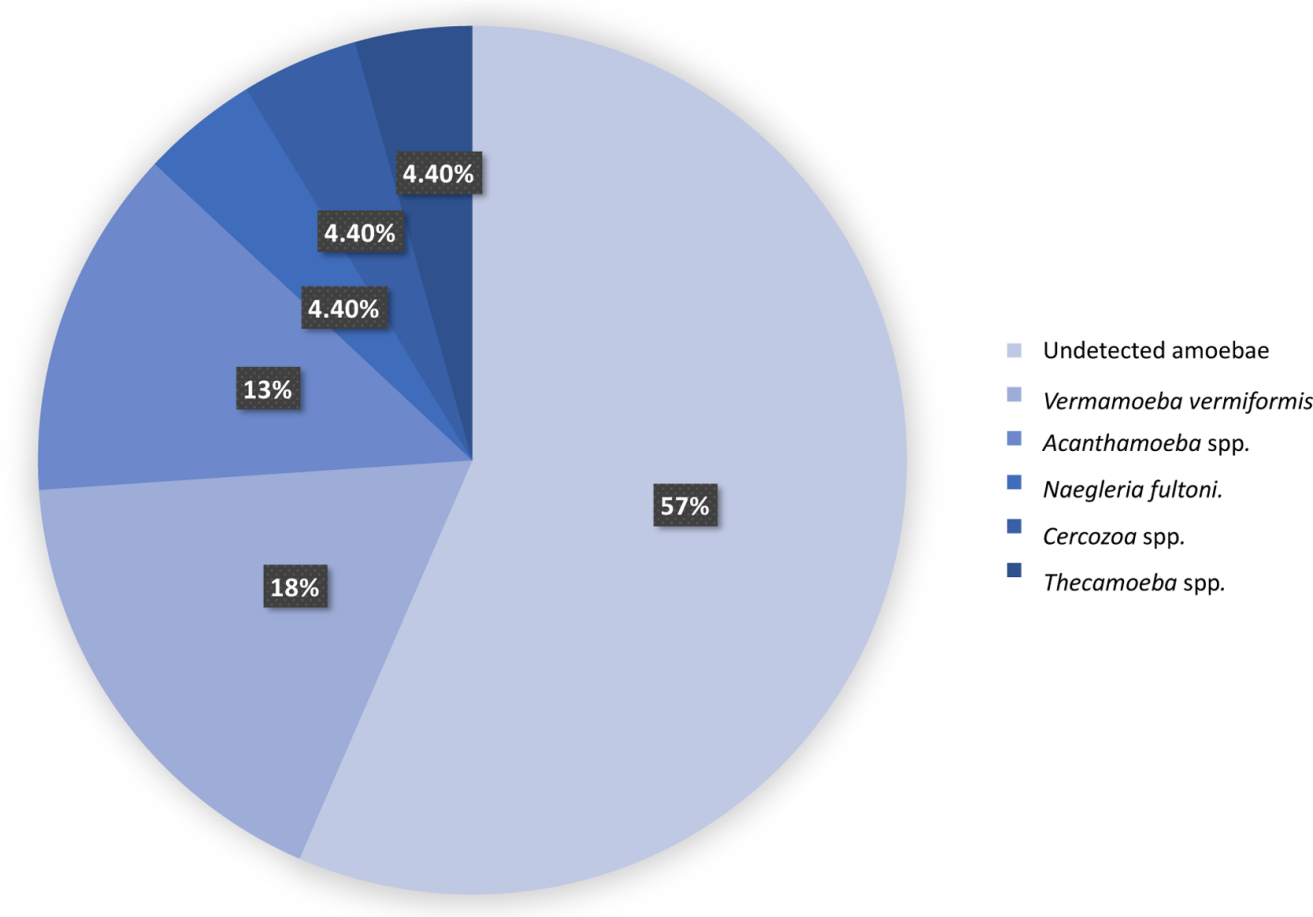
Distribution of FLA isolated in water samples of Tenerife.

**Figure 5:**
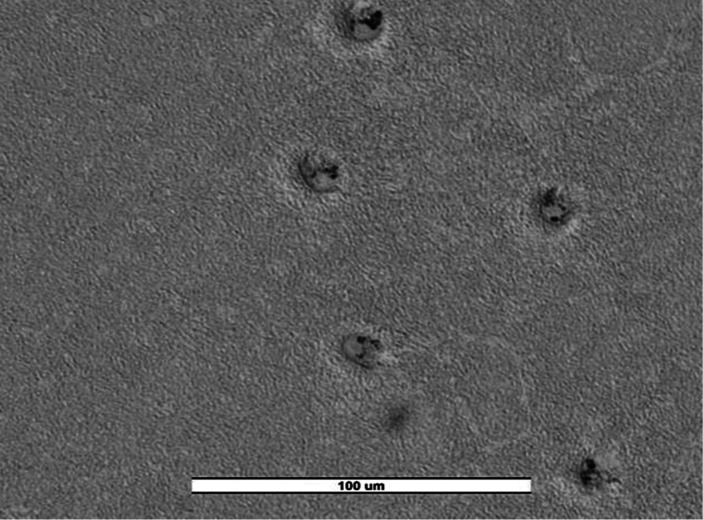
Trophozoites of Cercozoa spp. from an irrigation water sample on NNA (TFEW23). Image was obtained with an ECHO Revolution hybrid microscope (40×).

**Figure 6:**
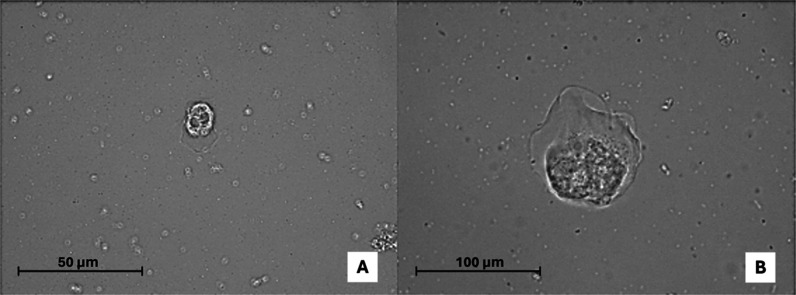
Trophozoites of Thecamoeba spp. at 40× (A) and at 100× (B) from soil and water samples (TFES14 and TFEW2). Images were obtained with a DM100 LED microscope.

The obtained sequences in the present study have been deposited in the GenBank database under the following accession numbers: PP339673 to PP339707 and PP413620 to PP413621. All of them presented >95% of homology with the available DNA sequences in this database.

The phylogenetic relationship of the FLA strains isolated in soil and water samples is represented in Figures 7 and 8, respectively. The tree of soil samples with the highest log likelihood (−2066.13) is shown. Initial tree(s) for the heuristic search were obtained automatically by applying neighbor-joining and BioNJ algorithms to a matrix of pairwise distances estimated using the Tamura-Nei model and then selecting the topology with superior log likelihood value. The percentage of replicate trees in which the associated taxa clustered together in the bootstrap test (500 replicates) is shown above the branches (next to the branches). This analysis involved 36 nucleotide sequences. There were a total of 302 positions in the final data set.

**Figure 7:**
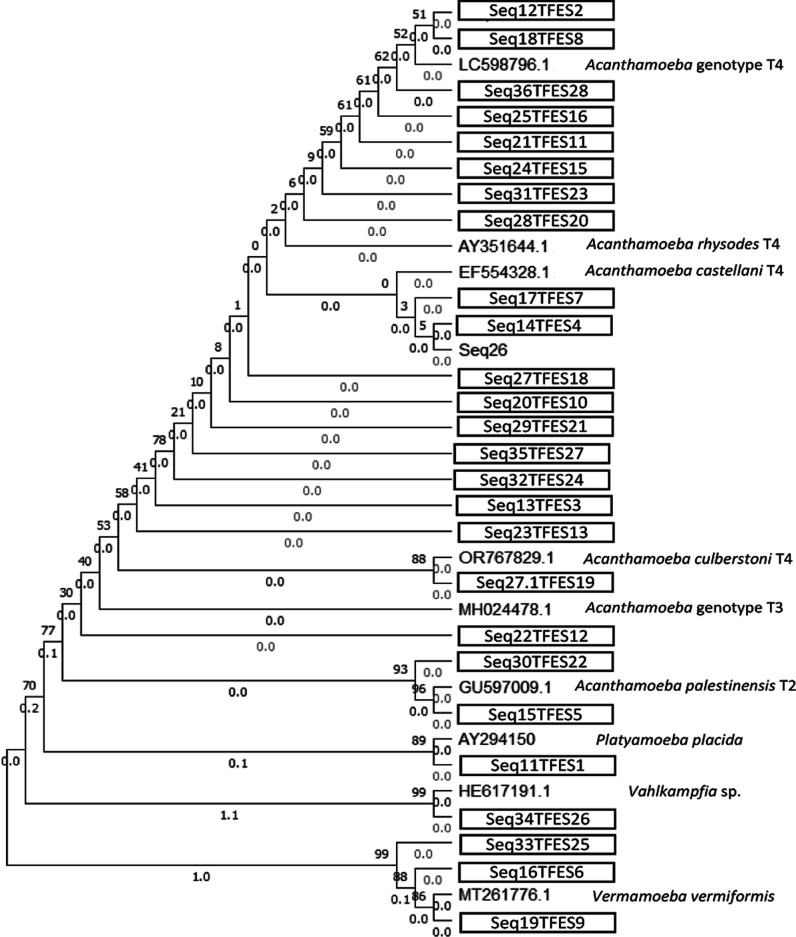
Phylogenetic connections among the FLA strains obtained from soil samples in this study. The isolates obtained in this study are marked within boxes.

**Figure 8:**
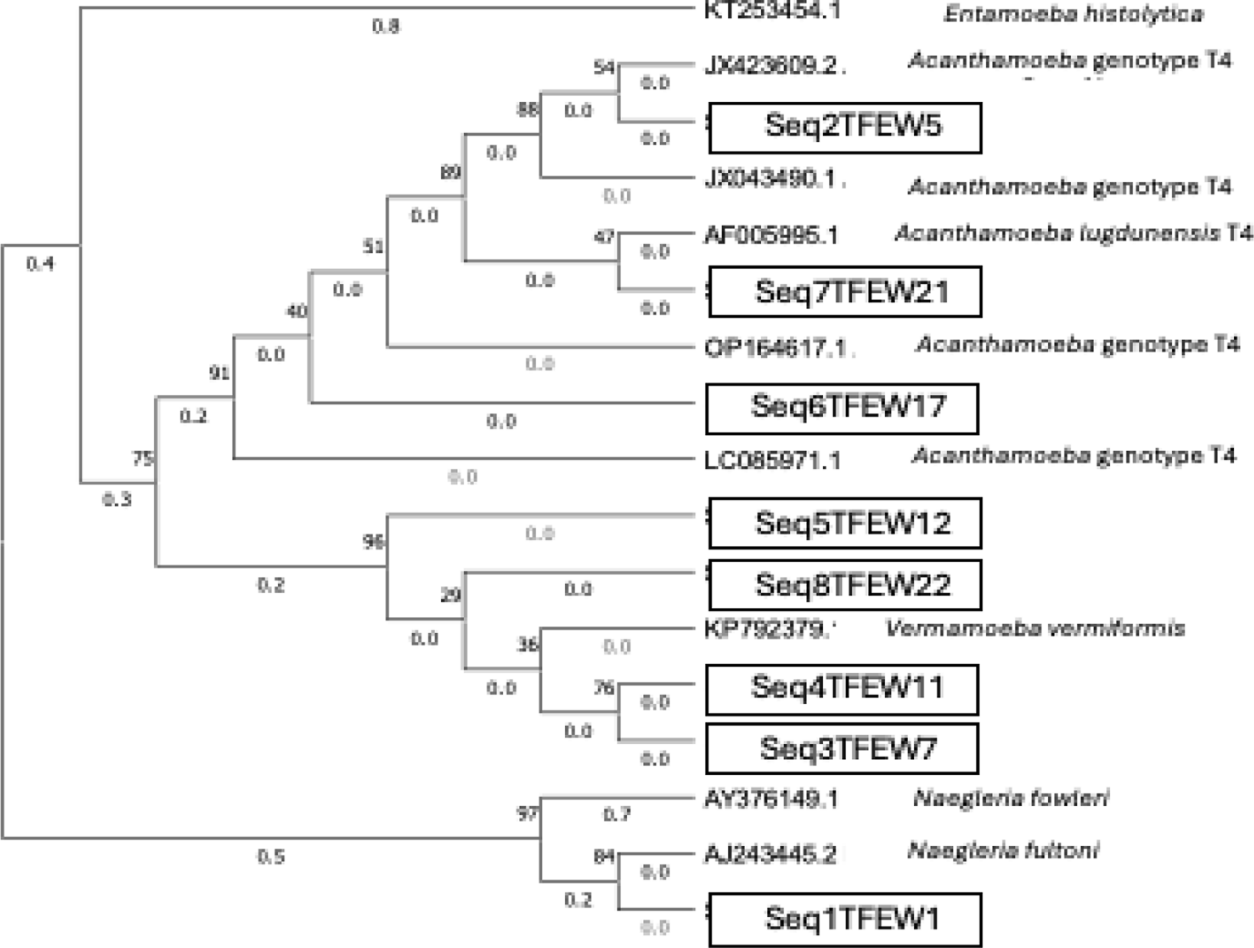
Phylogenetic relationship of the FLA strains isolated from water samples in the present study. The isolates obtained in the present study are identified in boxes.

The evolutionary history was inferred using the neighbor-joining method. The percentage of replicate trees in which the associated taxa clustered together in the bootstrap test (500 replicates) is shown next to the branches (next to the branches). The evolutionary distances were computed using the maximum composite likelihood method and are given in units of the number of base substitutions per site. This analysis involved 17 nucleotide sequences. All ambiguous positions were removed for each sequence pair (pairwise deletion option). There were a total of 844 positions in the final data set. Evolutionary analyses were conducted in MEGA11.

## Discussion

In the current investigation, the incidence of FLA in soils and water from human-related environments, such as school gardens, tap water, and recreational fountains, among others, was assessed. These protists, which are widely distributed, significantly increase the microbial pollution of water supplies.^29^ In fact, it has been noted that FLA contamination of water is likely to be a significant health issue due to their capacity of harboring other microorganisms that could be potentially pathogenic to humans.^30–32^

Considering that these FLAs represent a risk to both the environment and human health, different authors have reported their presence in a multitude of environments. Previously, Lorenzo-Morales et al. (2005) focused on demonstrating the presence of Acanthamoeba in soils and waters of Tenerife Island. In fact, in this study, the water samples collected were tap water and seawater, while among the tested soils, even beach sand was included. This previous study called for further investigations to determine the importance of these potential pathogens in the region.^17,18^ For this reason, we aimed to broaden the type of samples by collecting waters from recreational fountains, irrigation water, tap water, among others, soils from school gardens, and vegetable patches to explore a wider variety of FLA, including species beyond Acanthamoeba. However, tap waters were collected from the same municipalities, except for a few new ones included, and in particular, the recent study highlights the finding of V. vermiformis. Interestingly, this species is reported for the first time in tap water sources of Tenerife. Colonizing this water system could cause keratitis among contact lens wearers who use tap water instead of disinfectant to maintain their lenses.^34^ In line with sources, V. vermiformis is a ubiquitous and thermotolerant amoeba that is among the most common FLA. It shows an ability to spread disease but has also been associated with harmful bacteria.^35,36^ Therefore, this current study expands the search for new FLA.

The genus Acanthamoeba was the most frequently isolated in all environmental samples and is the most pathogenic and the most common FLA group. In addition, it is the genus best known to produce pathology in humans.^37^ The majority of Acanthamoeba species can cause cerebral infections as GAE, Acanthamoeba keratitis (AK), and less frequently skin lesions.^33,38^ Based on 18S rDNA sequencing, this genus has at least 23 genotypes (T1–T23).^39–41^ According to research conducted thus far, 90% of Acanthamoeba isolates that cause infections belong to the T4 genotype. Hence, the high prevalence of T4 isolates in infections is most likely caused by their higher virulence, characteristics that increase their transmissibility, and decreased susceptibility to chemotherapeutic agents.^42–44^ Despite the T4 genotype being the most frequently identified in human infection cases, it is also noteworthy in environmental samples.^45,46^ Our results supported these earlier reports. The molecular assays used in this investigation have allowed us to identify T2 (TFES5 and TFES22) and T3 (TFES12) genotypes in the soils from San Cristóbal de La Laguna included in this study. Omaña-Molina et al. have described that the T3 genotype is a causative agent of amoebic keratitis, and in this case, it affected only one eye of the patient.^47^ However, it is the second most prevalent genotype on three continents.^48^ Meanwhile, along with the T3 genotype, the T2 genotype (T2B) has also been isolated worldwide from patients with AK. Furthermore, it has less commonly been associated with GAE.^49,50^

As with many other FLA, Platyamoeba placida, Cercozoa spp., and Thecamoeba spp. were isolated in the present research work. Cercozoa is a phylum of nonpathogenic protists that is predominant in terrestrial systems and belongs to the Percolozoa (Heterolobosea) class and Rhizaria subgroup.^51,52^ Moreover, these free-living protozoa may help the spread of other pathogen populations, serving as potential hosts for endosymbiotic bacteria.^53,54^ Regarding Thecamoeba genus, it presents a widespread distribution in the environment and can be found in freshwater and marine habitats, soil, leaf litter, and plant surfaces, especially dead grass debris.^55^ In the current study, it was detected in water and soil samples (TFEW2 and TFES14).

A recent research showed that several Vahlkampfia species belonging to the family Vahlkampfiidae could potentially cause keratitis.^56^ Although Acanthamoeba spp. are the primary cause of amoebic keratitis, there have been various reported cases of coinfections of Acanthamoeba and Vahlkampfia genera and/or Vahlkampfia-related corneal injury.^57–59^ In one of the studied soil samples (TFES26), Vahlkampfia spp. was isolated, posing a risk of eye infection during gardening activities in this vegetable patch. In the same family is the genus Naegleria with the only pathogenic species, N. fowleri.^60^ In the samples collected in Tenerife, the available data on this genus are scarce, with N. fultoni species being reported for the first time in the region.

Overall, the current study presents a high variability of FLA in both environmental sources on the island of Tenerife. There is a clear difference between the FLA isolated in the water and soil samples. Although the genus Acanthamoeba was isolated in water and soil samples, it has been more present in soils since it is one of its main habitats due to the abundance of nutrients, which provide a favorable environment for survival and proliferation.^37,44^ In contrast, V. vermiformis was more commonly identified in water samples, including tap water, recreational fountains, and freshwater fish tanks.^36^ This may be due to the ability of V. vermiformis to tolerate and proliferate in aquatic environments, especially those that are relatively controlled and temperature stable. The identification of N. fultoni and other amoebae in water sources also suggests that these aquatic environments may harbor a variety of amoebae that could pose a risk to human health, especially in recreational or domestic settings.

## Conclusions

The results obtained show that the distribution of FLA is influenced by the type of environment, which reinforces the need to adapt prevention and control strategies to minimize direct contact between humans and these protozoa. Considering that, in this study, a global prevalence of FLA of 74.50% was obtained.

## Supplementary Material



## Notes

Additional details and results, including tables showing the presence of FLA in each of the water and soil samples from Tenerife (PDF)
