# Supplementary material for: Isolation and Molecular Identification of Pathogenic Free-Living Amoebae from Environmental Samples in Tenerife, Canary Islands, Spain
Source: ACS ES T Water. 2025 May 2;5(6):2861–9. doi: 10.1021/acsestwater.4c00573 (PMC12172190; doi:10.1021/acsestwater.4c00573)
Supplement: Supplementary file 1 [file ew4c00573_si_001.pdf]

Isolation and molecular identification of pathogenic free-living amoebae from environmental samples in Tenerife, Canary Islands, Spain.

Patricia Pérez-Pérez[1,2*]; María Reyes-Batlle[1,2,3]; Rodrigo Morchón[4]; José E. Piñero[1,2,3] and Jacob Lorenzo-Morales[1,2,3].

[1] Instituto Universitario de Enfermedades Tropicales y Salud Pública de Canarias (IUETSPC), Universidad de La Laguna (ULL), Avenida Astrofísico Francisco Sánchez S/N, San Cristóbal de La Laguna, 38206, Tenerife, Spain

[2] Departamento de Obstetricia y Ginecología, Pediatría, Medicina Preventiva y Salud Pública, Toxicología, Medicina Legal y Forense y Parasitología, Facultad de Farmacia, Universidad de La Laguna, San Cristóbal de La Laguna, 38200, Tenerife, Spain

[3] Consorcio Centro de Investigación Biomédica en Red de Enfermedades Infecciosas (CIBERINFEC), Instituto de Salud Carlos III,Madrid, 28029 Spain

[4]Zoonotic Infections and One Health GIR, Laboratory of Parasitology, Faculty of Pharmacy, University of Salamanca, Salamanca, 37008,Spain

*Email:pperezpe@ull.edu.es

Table 2. FLA species isolated from the evaluated soil samples of Tenerife (NNA: FLA growth in non-nutrient agar culture; PCR: FLA detection by PCR; homology (%) related to NCBI Database sequence).

**Soil samples**						
Sample code	Locality	Soil type	NNA	PCR	Genus/species	Homology (%)
TFES1	Tegueste	School garden	+	+	*Platyamoeba placida*	≥ 95%
TFES2	Tegueste	School garden	+	+	*Acanthamoeba castellanii* T4	≥ 95%
TFES3	La Laguna	School garden	+	+	*Acanthamoeba* sp. T4	≥ 95%
TFES4	La Laguna	School garden	+	+	*Acanthamoeba* sp. T4	≥ 95%
TFES5	La Laguna	School garden	+	+	*Acanthamoeba palestinensis* T2	≥ 95%
TFES6	La Laguna	School garden	+	+	*Vermamoeba vermiformis*	≥ 95%
TFES7	La Laguna	School garden	+	+	*Acanthamoeba rhysodes* T4	≥ 95%
TFES8	La Laguna	School garden	+	+	*Acanthamoeba* sp. T4	≥ 95%
TFES9	La Laguna	School garden	+	+	*Vermamoeba vermiformis*	≥ 95%
TFES10	La Laguna	School garden	+	+	*Acanthamoeba rhysodes* T4	≥ 95%
TFES11	La Laguna	School garden	+	+	*Acanthamoeba castellanii* T4	≥ 95%
TFES12	La Laguna	School garden	+	+	*Acanthamoeba* sp. T3	≥ 95%
TFES13	La Laguna	School garden	+	+	*Acanthamoeba* sp. T4	≥ 95%
TFES14	La Laguna	School garden	+	-	*Thecamoeba* spp.	
TFES15	La Laguna	School garden	+	+	*Acanthamoeba* sp. T4	≥ 95%
TFES16	La Guancha	Vegetable patch	+	+	*Acanthamoeba* sp. T4	≥ 95%
TFES17	La Guancha	Vegetable patch	+	+	*Acanthamoeba* sp. T4	≥ 95%
TFES18	La Victoria de Acentejo	Vegetable patch	+	+	*Acanthamoeba castellani* T4	≥ 95%

TFES19	Icod de los Vinos	Vegetable patch	+	+	*Acanthamoeba culbertsoni* T4	≥ 95%
TFES20	Icod de los Vinos	Vegetable patch	+	+	*Acanthamoeba* sp. T4	≥ 95%
TFES21	Icod de los Vinos	Vegetable patch	+	+	*Acanthamoeba* sp. T4	≥ 95%
TFES22	Icod de los Vinos	Vegetable patch	+	+	*Acanthamoeba* sp. T2	≥ 95%
TFES23	Icod de los Vinos	Vegetable patch	+	+	*Acanthamoeba* sp. T4	≥ 95%
TFES24	Icod de los Vinos	Vegetable patch	+	+	*Acanthamoeba* sp. T4	≥ 95%
TFES25	Icod de los Vinos	Vegetable patch	+	+	*Vermamoeba vermiformis*	≥ 95%
TFES26	Icod de los Vinos	Vegetable patch	+	+	*Vahlkampfia* sp.	≥ 95%
TFES27	Icod de los Vinos	Vegetable patch	+	+	*Acanthamoeba* sp. T4	≥ 95%
TFES28	Icod de los Vinos	Vegetable patch	+	+	*Acanthamoeba* sp. T4	≥ 95%

Table 3. FLA species isolated from the evaluated water sources in Tenerife (NNA: FLA growth in non-nutrient agar culture; PCR: FLA detection by PCR; homology (%) related to NCBI Database sequence).

Water samples						
Sample code	Locality	Water type	NNA	PCR	Genus/species	Homology (%)
TFEW1	La Laguna	Recreational fountain	+	+	*Naegleria fultoni*	≥ 95%
TFEW2	La Laguna	Recreational fountain	+	-	*Thecamoeba* spp.	
TFEW3	Santa Cruz	Recreational fountain	-	-		
TFEW4	Santa Cruz	Recreational fountain	-	-		
TFEW5	Santa Cruz	Recreational fountain	+	+	*Acanthamoeba* sp. T4	≥ 95%
TFEW6	La Laguna	Recreational fountain	-	-		
TFEW7	Arafo	Recreational fountain	+	+	*Vermamoeba vermiformis*	≥ 95%
TFEW8	La Laguna	Recreational fountain	-	-		
TFEW9	La Laguna	Recreational fountain	-	-		
TFEW10	La Esperanza	Tap water	-	-		
TFEW11	San Isidro	Tap water	+	+	*Vermamoeba vermiformis*	≥ 95%
TFEW12	La Matanza	Tap water	+	+	*Vermamoeba vermiformis*	≥ 95%
TFEW13	Candelaria	Tap water	-	-		
TFEW14	La Victoria de Acentejo	Tap water	-	-		
TFEW15	Santa Cruz	Tap water	-	-		
TFEW16	La Orotava	Tap water	-	-		
TFEW17	La Laguna	Tap water	+	+	*Acanthamoeba* sp. T4	≥ 95%
TFEW18	La Laguna	Tap water	-	-		
TFEW19	Güimar	Tap water	-	-		
TFEW20	La Laguna	Tap water	-	-		
TFEW21	Santa Cruz	Ravine	+	+	*Acanthamoeba lugdunensis* T4	≥ 95%

TFEW22	La Laguna	Fish tank	+	+	*Vermamoeba vermiformis*	≥ 95%
TFEW23	La Laguna	Irrigation water	+	-	*Cercozoa* spp.	
